# Necrology

**Published:** 1898-06

**Authors:** 


### NECROLOGY.

Veterinarian P. M. McArthur died in Hot Springs, Ark. Buried in Winston, N. C., March 24th. A host of friends mourn his death.

